# Seroepidemiology of varicella-zoster virus in Korean adolescents and adults using fluorescent antibody to membrane antigen test

**DOI:** 10.1017/S0950268814002441

**Published:** 2014-09-19

**Authors:** S. B. HAN, K. R. KANG, D. H. HUH, H. C. LEE, J. H. KIM, J. H. KANG, S. H. MA

**Affiliations:** 1 Department of Pediatrics, College of Medicine, The Catholic University of Korea, Seoul, Republic of Korea; 2 Vaccine Bio Research Institute, College of Medicine, The Catholic University of Korea, Seoul, Republic of Korea; 3 Department of Pediatrics, Changwon Fatima Hospital, Changwon, Republic of Korea

**Keywords:** Epidemiology, fluorescent antibody to membrane antigen test, Korea, varicella-zoster virus

## Abstract

We conducted a cross-sectional seroepidemiological study in 2012–2013 to determine the seroprevalence of varicella-zoster virus (VZV) in adolescents and adults living in Korea, where varicella vaccination has been recommended universally at age 12–15 months since 2005. Residual serum samples were collected from 1196 healthy adults and adolescents aged ⩾10 years between November 2012 and March 2013. The fluorescent antibody to membrane antigen (FAMA) test and enzyme-linked immunosorbent assay (ELISA) were performed to determine the seroprevalence of VZV. The seroprevalences of VZV were compared between six age groups: 10–19, 20–29, 30–39, 40–49, 50–59, and ⩾60 years. The seroprevalence of VZV in the entire study cohort was 99·1% according to the FAMA test and 93·1% as determined by ELISA. The seroprevalences of the six age groups were as follows: 96·0%, 99·5%, 99·5%, 99·5%, 100%, and 100%, respectively, by the FAMA test, and 83·3%, 93·0%, 93·0%, 97·5%, 94·5%, and 97·5%, respectively, by ELISA. Seroprevalence increased significantly with age (*P* < 0·001); moreover, the seroprevalence in subjects aged 10–19 years was significantly lower than in other age groups (*P* < 0·001), as measured by both the FAMA test and ELISA. Thus, strategies to increase protective immunity against VZV in teenagers are necessary.

## INTRODUCTION

Varicella-zoster virus (VZV) is categorized as an alpha-herpes virus [1]. Primary infection by VZV causes chickenpox, and then latent infection is maintained in the dorsal root ganglia and cranial nerve nuclei [1, 2]. Infected individuals may experience herpes zoster or subclinical VZV reactivation by reactivation of latent VZV [1]. In particular, the frequency and severity of VZV reactivation are higher in elderly and immunocompromised patients, due to their decreased VZV-specific T-cell immunity [1, 3–6].

The prevalence of herpes zoster appears to be on the rise in Korea [7]; this trend is thought to be due to a corresponding increase in the proportion of elderly and immunocompromised patients, such as patients with diabetes, end-stage renal disease, cancer and solid organ or haematopoietic cell transplantation recipients [8–11]. Further potential reasons for these increases include changes in the society, environment and in the virus [12]. However, much is still unknown regarding why herpes zoster incidence is increasing; therefore, further studies are needed to examine this question. Varicella vaccination was introduced in Korea in 1988, and the Korean National Immunization Program has recommended only one mandatory dose of varicella vaccination at age 12–15 months since 2005 [13]. Although the vaccine coverage rate for varicella was reported to be 96·8% in children aged <6 years in 2008 in Korea [14], breakthrough VZV infection (VZV infection in persons who previously received varicella vaccination) can still occur in Korea and the occurrence of herpes zoster in adults may be on the rise [15, 16]. Such breakthrough infections have also been observed in other countries where varicella vaccination has been recommended [17–19]. For early detection of such epidemiological and seroepidemiological changes, and in order to establish appropriate strategies for controlling breakthrough VZV infections, repeated surveillance of immune status against VZV in the community is necessary. However, only studies including limited numbers of subjects have been performed in Korea [20–22]. In addition, only one seroepidemiological study for VZV using the fluorescent antibody to membrane antigen (FAMA) test has been performed; this study was limited to adults aged >40 years [21]. Although enzyme-linked immunosorbent assay (ELISA) can be easily performed compared to the FAMA test, several studies have reported the FAMA test to have higher sensitivity in determining protective immunity against VZV in cross-sectional and vaccine efficacy studies [21, 23–27]. Therefore, the FAMA test is recognized as the gold standard for determining VZV immunity [28].

We conducted this cross-sectional and age-stratified seroepidemiological study of VZV using the FAMA test to determine the current VZV seroprevalence in Korea, where a single dose of varicella vaccination is recommended. Because the FAMA test is such a labour- and time-intensive test to perform routinely in clinical practice, we evaluated the usefulness of ELISA again. The results of the FAMA test and a commercially available ELISA kit were compared.

## MATERIALS AND METHODS

### Collection of residual serum samples

Between November 2012 and March 2013, residual serum samples were collected from healthy adults and adolescents aged ⩾10 years who visited the Health Promotion Centres of three hospitals in Korea. This study was approved by the Institutional Review Board (IRB) of each participating hospital, and the need for informed consent was waived (IRB no. KC12TSSI0721).

### FAMA test

VZV-infected cells were provided from the Mogam Biotechnology Institute (Yongin, Korea). MGLu human embryonic lung fibroblast cells were infected with VZV strain Mogam (MAV/06), and collected when the cytopathic effect was observed in 70–80% of the cells.

The FAMA test was performed at the Vaccine Bio Research Institute, College of Medicine, The Catholic University of Korea (Seoul, Korea) as described previously [29], but with slight modification. In brief, 20 *μ*l (2 × 10^5^ cells/ml) of the VZV-infected cell suspension was aliquoted into each well of a 5-mm 12-well slide, and the cells were incubated overnight in a humidity chamber. The slide was then dried aseptically in a 45 °C dry bath, and then fixed in cold acetone. After washing with phosphate-buffered saline (PBS), the slide was air-dried at room temperature. Fixed cells were stored at −70 °C until the FAMA test was performed. Aliquots of residual serum were inactivated for 30 min at 56 °C and then serially diluted twofold in PBS, resulting in final dilutions of 1:2 to 1:256. Serum dilutions (20 *μ*l) were aliquoted into each well of the acetone-fixed slide and then incubated for 30 min at 37 °C. After three washes with PBS, slides were air-dried at room temperature. Next, 20 *μ*l fluorescein-5-isothiocyanate (FITC)-conjugated anti-human IgG antibodies (polyclonal rabbit anti-human IgG/FITC, Dako Denmark A/S, Denmark), which was diluted 1:50 in PBS, was added to each well; slides were then incubated for 30 min at 37 °C. Cells were washed three times with PBS and then were air-dried at room temperature, mounted with ClearMount™ Mounting Solution (Invitrogen, USA), and covered with a coverslip. Using a fluorescent microscope (100× magnification), the incubated cells were examined for complete ring-like fluorescence around their surfaces; this process was performed by two independent investigators.

**Fig. 1.**
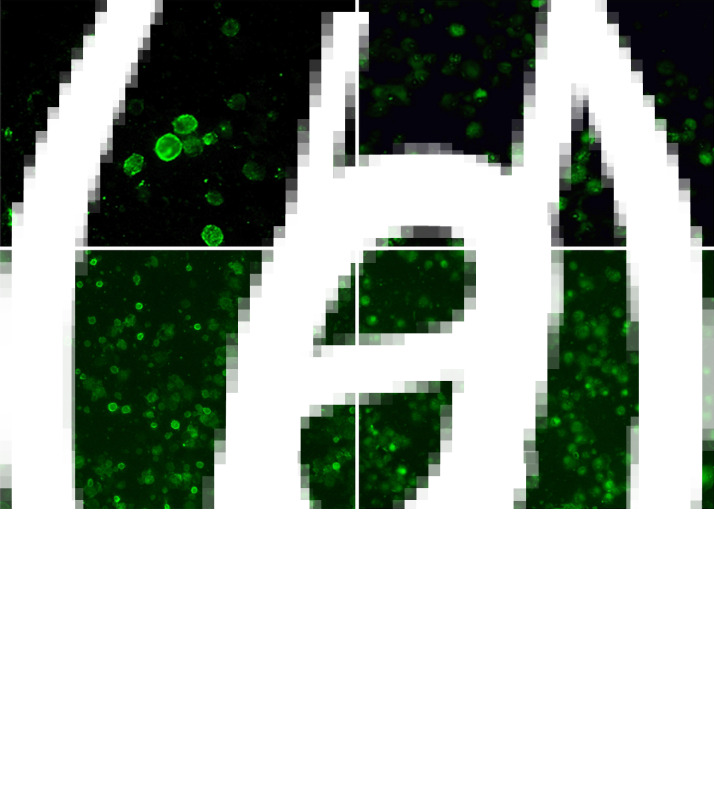
Microscopy of varicella-zoster virus-infected cells used in the fluorescent antibody to membrane antigen test. (*a*) Positive control (200× magnification), (*b*) negative control (200× magnification), (*c*) positive result (100× magnification) and (*d*) negative result (100× magnification).

Anti-VZV antibodies (National Institute for Biological Standards and Control, UK) were used as a positive control. Serum obtained from a child without any previous history of chickenpox or varicella vaccination was obtained from the Mogam Biotechnology Institute, and was used as a negative control (Fig. 1). FAMA titres of ⩾1:4 were considered VZV positive (Fig. 1). Since a FAMA titre ⩾1:16 has been reported to indicate full protection against VZV infection [30, 31], we defined FAMA titres ⩾1:16 as strongly positive and FAMA titres between 1:2 and 1:8 as weakly positive.

### Whole virus ELISA

Anti-VZV IgG antibodies were measured using a commercial whole-cell ELISA kit (Enzygnost^®^ Anti-VZV/IgG ELISA kit, Siemens Healthcare Diagnostics, Germany), which was based on an indirect enzyme immunoassay, in accordance with the manufacturer's recommendation. Assay results were characterized qualitatively, based on their difference of absorbance values (Δ*A*). This difference was defined as the absorbance value of the tested serum minus the absorbance value of the negative control serum. Assay results were classified as follows: Δ*A* < 0·100, negative; 0·100 ⩽ Δ*A* ⩽ 0·200, equivocal; Δ*A* > 0·200, positive. Samples yielding an equivocal result were retested in duplicate.

### Data analysis and statistical analysis

Study subjects were divided into the following six age groups: group 1 (10–19 years), group 2 (20–29 years), group 3 (30–39 years), group 4 (40–49 years), group 5 (50–59 years), and group 6 (⩾60 years). Seropositivity was defined as testing strongly or weakly positive in the FAMA test and positive by ELISA. The seroprevalence of each age group, as determined by the FAMA test and ELISAs, were compared. In addition, the proportion of subjects testing strongly positive, weakly positive and negative in the FAMA test were also compared in the six age groups. The degree of agreement between the FAMA test results and ELISA results was also determined. A *χ*^2^ test was used to compare the results of different age groups, and the kappa (*κ*) value was employed to evaluate the degree of correlation between the FAMA test results and the ELISA results. The *κ* value is interpreted as follows: *κ* < 0·00: poor correlation; 0·00 ⩽ *κ* ⩽ 0·20: slight correlation; 0·21 ⩽ *κ* ⩽ 0·40: fair correlation; 0·41 < *κ* ⩽ 0·60: moderate correlation; 0·61 < *κ* ⩽ 0·80: substantial correlation; and *κ* > 0·80: almost perfect correlation [32]. Statistical analysis was performed with SPSS Statistics v. 17·0 (SPSS Inc., USA), and statistical significance was defined as a two-tailed *P* value <0·05.

## RESULTS

During the study period, residual serum samples were collected from 1196 adolescents and adults, including 602 males and 594 females (Table 1). The number of subjects in each age group ranged from 198 to 200; no significant differences were observed in the sex distribution between age groups (Table 1).

**Table 1.** Distribution of the sexes of the subjects enrolled in this study according to age group

Sex	Group 1	Group 2	Group 3	Group 4	Group 5	Group 6	Total
Male, *n* (%)	102 (51·5)	100 (50·3)	100 (50·0)	100 (50·0)	100 (50·3)	100 (50·0)	602 (50·3)
Female, *n* (%)	96 (48·5)	99 (49·7)	100 (50·0)	100 (50·0)	99 (49·7)	100 (50·0)	594 (49·7)
Total, *n* (%)	198 (100·0)	199 (100·0)	200 (100·0)	200 (100·0)	199 (100·0)	200 (100·0)	1196 (100·0)

### Fluorescent antibody to membrane antigen test results

**Fig. 2.**
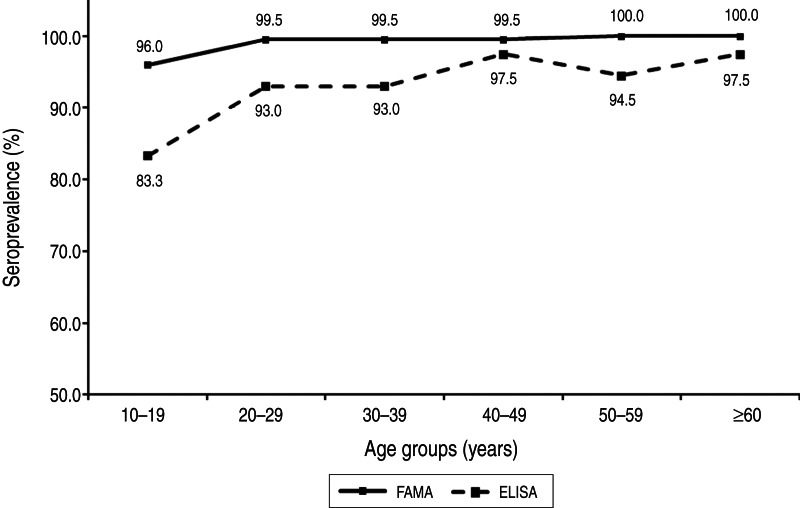
Seroprevalences as determined by the fluorescent antibody to membrane antigen (FAMA) test and enzyme-linked immunosorbent assay (ELISA).

**Fig. 3.**
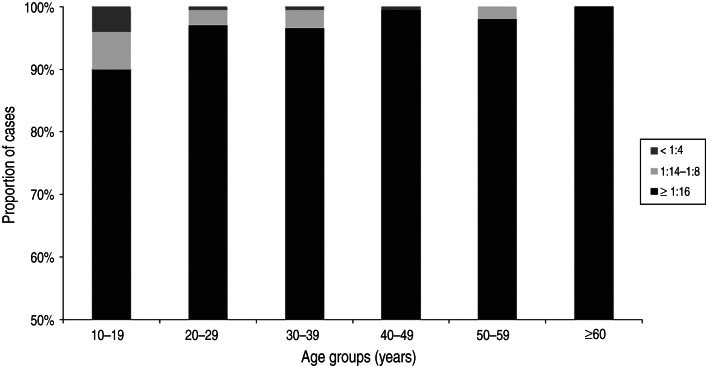
Proportion of cases according to the fluorescent antibody to membrane antigen titres in each age group.

Eleven (0·9%) of the 1196 subjects were VZV-negative and 1185 (99·1%) were positive according to the FAMA test (Table 2). Of the subjects who were positive on the FAMA test, only four (0·3%) subjects showed weakly positive results; the remaining 1181 (98·8%) subjects had strongly positive titres of ⩾1:16 (Table 2). The seroprevalences of groups 1–6 were 96·0% (190/198), 99·5% (198/199), 99·5% (199/200), 99·5% (199/200), 100·0% (199/199) and 100·0% (200/200), respectively (Fig. 2). The seroprevalence of VZV tended to increase with age (*P* < 0·001), and group 1 (10–19 years) had a significantly lower seroprevalence compared to the older age groups (*P* < 0·001). The proportion of strongly positive subjects also increased significantly with age (*P* < 0·001), and all of the subjects in group 6 (⩾60 years) had strongly positive results (Fig. 3).

**Table 2.** Degree of agreement between the results obtained using the FAMA test and ELISA

ELISA result	FAMA titre		
	<1:4 (negative)	1:4–1:8 (weakly positive)	⩾1:16 (strongly positive)
Negative	9 (81·8)	3 (75·0)	12 (1·0)
Equivocal	2 (18·2)	1 (25·0)	55 (4·7)
Positive	0 (0·0)	0 (0·0)	1114 (94·3)
Total	11 (100·0)	4 (100·0)	1181 (100·0)

ELISA, Enzyme-linked immunosorbent assay; FAMA, fluorescent antibody to membrane antigen.

### ELISA results

Twenty-four (2·0%), 58 (4·9%) and 1114 (93·1%) of the 1196 subjects showed negative, equivocal and positive ELISA results, respectively (Table 2). A total of 66 subjects exhibited equivocal results on the initial ELISA test; however, eight of these had positive results on repeated testing. The seroprevalences of groups 1–6 were 83·3% (165/198), 93·0% (185/199), 93·0% (186/200), 97·5% (195/200), 94·5% (188/199) and 97·5% (195/200), respectively (Fig. 2). The seroprevalence determined by ELISA also tended to increase with age (*P* < 0·001), and group 1 (10–19 years) showed a significantly lower seroprevalence compared to the older age groups (*P* < 0·001).

### Degree of agreement between the results of FAMA test and ELISA

Nine (0·8%) subjects were VZV negative according to both the FAMA test and ELISA, and 1114 (93·1%) were strongly positive according to the FAMA test and positive by ELISA (Table 2). In addition, one (0·1%) subject had a weakly positive result in the FAMA test and an equivocal result by ELISA (Table 2). The FAMA test and ELISA showed agreement in 94·0% of all subjects, and the two tests showed a fair agreement (*κ* = 0·247, *P* < 0·001). ELISA demonstrated 94·0% sensitivity and 100% specificity, in addition to yielding a 100% positive predictive value and a 13·4% negative predictive value, when the FAMA test was applied as the gold standard.

## DISCUSSION

In this study, the VZV seroprevalence in Korean adolescents and adults was evaluated using the FAMA test. Most previous reports using the FAMA test employed cells infected either with the Oka strain of VZV, or with clinical strains obtained from patients with chickenpox or herpes zoster, as antigens [21, 27, 29, 33–35]; however, this study is the first to use cells infected with the MAV/06 strain of VZV as an antigen. The VZV MAV/06 strain is an attenuated VZV strain used in the production of varicella vaccines; the original clinical strain was obtained from a child with chickenpox in 1989 in Seoul, Korea [36]. The MAV/06 strain is genetically similar to the Oka strain, and is classified in clade 2 along with other varicella vaccine strains, such as the VarilRix and VariVax strains [36]. The MAV/06 strain may be the most appropriate VZV strain for use in the FAMA test to determine seroprevalence in Koreans because the MAV/06 strain originated from a Korean clinical strain and a varicella vaccine manufactured from the MAV/06 strain (SuduVax^®^, Green Cross Corporation, Korea) has been administered mainly to Korean children since 1994. Considering the finding that FAMA tests using clinical VZV strains of several genotypes as FAMA antigens still elicited appropriate antibody responses in subjects who received varicella vaccines with the Oka strain [30] and the MAV/06 strain is genetically very similar to the Oka strain [36], the MAV/06 strain can also presumably be used in FAMA tests to evaluate the immunogenicity of vaccines based on the Oka strain. SuduVax has been exported to several countries in Central America and Southeast Asia since 2000, and so the FAMA test using the MAV/06 strain may also be useful in those countries.

In accordance with a previous report [21], the FAMA test yielded higher seroprevalences than the ELISA in the present study. Although the results of these two tests showed only fair agreement, our results support the use of ELISA as a screening test. In our study, ELISA showed high specificity and sensitivity; moreover, ELISA is a much less labour- and time-intensive test compared to the FAMA test. However, ELISA may be less useful in the future when a greater portion of the population has been vaccinated. ELISA had lower sensitivity compared to the FAMA test in determining protective immunity against VZV, especially in vaccinees [25–27, 37], and thus the false-negative rate of ELISA may increase with the growing vaccinated population.

The VZV seroprevalences obtained by the FAMA test in the present study were quite high compared to previous seroepidemiological studies in Korea and Germany [21, 35]. However, the seroprevalence in our study was significantly lower in group 1 (10–19 years) than in any of the other age groups; moreover, the proportion of subjects yielding strongly positive results, which were indicative of full protection against VZV infection, was also significantly lower in group 1. The seroprevalences determined by ELISA showed a similar trend by age group. A previous study, using the same commercial ELISA kit used in the present study, was performed in 2009 in Korea; that study reported a VZV seroprevalence of ~94% in teenage subjects and 98% in subjects aged ⩾20 years [22]. In the present study, the seroprevalences determined by ELISA were lower than they were in a previous report, 83·3% in teenage subjects and 93·0% in subjects in their twenties and thirties. This decreased seroprevalence may be caused by the loss of vaccine-induced immunity, which can occur within a decade after varicella vaccination [18]. The introduction of childhood varicella vaccination particularly reduced the incidence in children [18, 38]; however, childhood varicella vaccination may also reduce the exogenous booster effect acquired by community exposure to VZV, which may explain the lower seroprevalence of VZV in teenage subjects. Although mandatory varicella vaccination has been recommended since 2005 in Korea, a single dose of varicella vaccination at age 12–15 months has been performed in accordance with parental preference since 1988. The vaccine coverage rate for varicella was reported to be 72·5% in one province of Korea in 2000 [39]. Considering the vaccine coverage rate prior to 2005, the effect of childhood varicella vaccination on VZV seroprevalence may be greater than we expected, and the decreased seroprevalence observed in subjects in their twenties and thirties indicates that the effects of these seroprevalence-reducing factors might extend far beyond the teenage years. Moreover, variation in the regions where serum samples were collected and in the age-matched sample size between the former study [22] and the present study may contribute to the seroprevalence variation seen in adolescents and young adults.

In the USA, breakthrough varicella and varicella outbreaks continued to be reported after the introduction of one-dose varicella vaccination programme, with 15–20% of one-dose vaccinees developing breakthrough varicella [40]. Accordingly, a second dose of varicella vaccination was recommended for children at age 4–6 years in the USA since 2006 based on the data showing a higher vaccine efficacy and lower rates of breakthrough disease in two-dose vaccinees compared to one-dose vaccinees [40]. In Korea, where single-dose varicella vaccination is recommended, the exact VZV seroprevalence should be determined using the FAMA test, and surveillance for breakthrough VZV infection should be performed. The necessity of booster varicella vaccination should be decided based on the observed seroprevalence and breakthrough infection rate of VZV.

Although adults aged >50 years exhibited 100% VZV seroprevalence in the FAMA test, the prevalence of herpes zoster is on the rise in the Korean elderly [7]. This observation emphasizes the important role of cellular immunity in preventing herpes zoster in the elderly. Accordingly, developing a strategy to enhance cellular immunity against VZV is of particular importance to the elderly; a zoster vaccination for adults could be one effective method.

Our study does have some limitations. For instance, it did not include children aged <10 years because young children seldom visit a health promotion centre for just a routine check-up in Korea. Therefore we could not collect a sufficient amount of serum samples from children aged <10 years, and could not directly evaluate the effectiveness of the varicella vaccine given universally at age 12–15 months. In addition, serum samples in the present study were collected from selected areas of Korea without randomization, and therefore the seroepidemiological findings of this study may not reflect the serological status of the Korean population as a whole. Future investigations should be performed as a nationwide study, including all age groups. We did not collect clinical information for subjects, including their previous history of VZV infection and varicella or zoster vaccination, but their previous disease and vaccination status may affect their protective immunity against VZV. Although the FAMA test was performed in this study using the MAV/06 strain as a FAMA antigen, and a similarly high seroprevalence was obtained as was seen in the FAMA test using the Oka strain, we did not directly compare the results of FAMA tests using the MAV/06 strain and those using the Oka strain. Therefore, we could not determine which FAMA antigen provides more reliable results, and the applicability of the FAMA test with the MAV/06 strain to other studies evaluating the immunogenicity of vaccination with the Oka strain and patients' immune status against clinical strains should be further studied.

In conclusion, we found that the VZV seroprevalence in Korean adolescents and adults exceeded 95% in 2012, and that VZV seroprevalence tended to increase with age. In accordance with this observation, VZV seroprevalence was lower in teenage subjects, especially the proportion of strongly positive subjects; moreover, seroprevalence could be even lower in children aged <10 years. Thus, further seroepidemiological studies that include children and use the FAMA test should be conducted in order to determine the accurate VZV seroprevalence in Korea. The results of such studies will help determine the necessity of booster varicella vaccination in Korean children.
